# Mapping support systems: a cross-sectional examination of personal support networks, perceived support, mental health outcomes, and help-seeking behaviours among UK undergraduate students

**DOI:** 10.1186/s12889-025-24360-1

**Published:** 2025-09-30

**Authors:** Emily Vicary, Dharmi Kapadia, Penny Bee, Helen Brooks

**Affiliations:** 1 Division of Nursing, Midwifery and Social Work, School of Health Sciences, Manchester Academic Science Centre, University of Manchester, Manchester, UK; 2 Department of Sociology, University of Manchester, Manchester, UK

**Keywords:** Social support, Personal support networks, Undergraduate mental health, Help-seeking behaviour, University students, Perceived support availability, Network size, Mental health outcomes, Higher education

## Abstract

**Background:**

Undergraduate students in the UK experience elevated rates of mental health difficulties but often face barriers to accessing formal support. Personal support networks – the friends, family, and other social ties students rely on - may play a critical role in promoting good mental health and guiding effective help-seeking. This study aimed to investigate the relationship between support network structure, perceived support availability, and mental health outcomes and help-seeking intentions among UK undergraduate students.

**Methods:**

A cross-sectional survey was completed by 287 undergraduates from 68 UK universities. Participants reported the size of their support network, relationship durations, relationship diversity, and support diversity using a modified Perceived Support Network Inventory. Perceived support was assessed using the Multidimensional Scale of Perceived Social Support. Depression and anxiety were measured with the PHQ-9 and GAD-7, and help-seeking intentions with an adapted General Help-Seeking Questionnaire. Associations were examined using Spearman’s rank-order correlational analyses and four multiple linear regression models.

**Results:**

Larger, longer-standing, and more diverse networks were positively associated with higher levels of perceived support. Perceived support predicted less severe depression and anxiety symptoms, independent of network structure. Students with higher perceived support were more likely to seek informal help and less likely to seek formal help. Network structural features generally did not predict help-seeking, although greater support diversity was associated with lower intention to seek informal help.

**Conclusions:**

Perceived availability of support, rather than structural characteristics of support networks, was the primary factor linked to improved mental health and help-seeking preferences. Strengthening students’ perceived support may be critical for promoting resilience and encouraging appropriate help-seeking behaviour.

**Supplementary Information:**

The online version contains supplementary material available at 10.1186/s12889-025-24360-1.

## Introduction

Undergraduate students in the UK face a complex set of stressors – including academic demands, financial pressures, and social adjustment – that place them at elevated risk for mental health difficulties. High rates of anxiety, depression, and psychological distress are well documented in this group [1, 2], with adverse implications for academic performance, student retention, and long-term wellbeing [3]. Although some national surveys suggest students have lower rates of common mental health problems compared to non-student peers, the trajectory is steeper in higher education; year-on-year increases in anxiety and depressive symptoms underscore a growing burden within this higher-education context [4]. Social Network Theory (SNT) provides a useful lens here: when formal services are overstretched or difficult to access [5, 6], informal ties become the primary channels through which emotional, informational, and instrumental resources flow [7].

Building on SNT, Social Capital Theory (SCT) conceptualises those ties a form of “capital” [8]. Bonding capital – close, homogenous ties such as family or long-standing friends – offers depth and trust, whereas bridging capital – more diverse, cross-cutting ties – extends reach and range. Accordingly, we conceptualise students’ personal support networks as the main conduit through which emotional, informational, and practical resources are mobilised to shape mental health outcomes. In practical terms, this network encompasses friends, family members, romantic partners, and mentors that an individual can draw upon for assistance, companionship, or guidance [9]. As university life often involves repeated relocations and shifting identity, these networks are inherently dynamic, being dismantled and rebuilt across home, campus, and digital spaces [10].

Structural characteristics of support networks – such as size, relationship duration, relationship diversity, and support diversity – are theorised to influence the availability and breadth of support that is potentially available. Larger and more diverse networks are typically associated with access to a broader range of supportive resources [11, 12], and evidence suggests that greater network size, longer-standing relationships, and greater relationship diversity are linked to improved mental health and quality of life [13, 14]. SCT helps explain these patterns: bonding ties provide the trust that sustains emotional help, while bridging ties import new information or opportunities that might buffer stress. Yet structural expansion is not automatically beneficial. When relationships lack quality or reliability, even large and diverse networks can fail to deliver effective help [8, 15].

This disconnect is captured by the Stress-Buffering Model (SBM), which proposes that it is the perception that support will be available – rather than structural metrics per se – that moderates the impact of stress on mental health [16]. Such support encompasses multiple dimensions typically categorised in six key types: emotional, financial, instrumental, informational, appraisal, and social participatory support [16, 17]. Access to a broader range of support types can yield cumulative benefits for wellbeing; for example, appraisal support may bolster self-esteem, while instrumental assistance may meet practical needs. In the UK, students who can draw on diverse types of support generally reported better overall well-being than those relying predominantly on a single form of support [18]. This functional diversity may also shape students’ help-seeking preferences, particularly if specific support needs are consistently met or unmet within their personal networks.

Yet despite this functional complexity, structural and perceptual aspects of support are rarely analysed together. Few studies have tested whether structural advantages emphasised by SNT and SCT – such as greater size, longevity, or tie diversity – actually result in the internalised sense of support that the SBM identifies as psychologically protective. This leaves open the central empirical question of whether structure translates into perception in practice, particularly among UK-based undergraduates.

An emerging body of research does suggest that perceived social support – the belief that help will be available when needed – may exert a more substantial influence on mental health than structural composition alone [19]. Among students diagnosed with mental illnesses, a systematic review revealed that greater perceived social support is associated with lower levels of depression, anxiety, stress, and suicidal ideation [20]. However, much of the existing research has focused on clinical or international student sub-populations. Relatively few studies have examined how UK undergraduates perceive the support available to them, and fewer still have considered these perceptions in tandem with the structural features of students’ personal networks. Studies that do include both elements often isolate them analytically, even though they likely operate in interaction. As a result, there remains limited empirical understanding of how structural and perceptual features of support networks combine to shape mental health outcomes in UK student populations. Perceived support may therefore influence not only how students experience distress, but also how they navigate decisions around seeking help.

These same network features also have essential implications for help-seeking behaviour. As framed by SNT, personal networks shaped behavioural pathways by structuring the flow of information, trust, and norms around help-seeking. Strong, high-quality relationships can encourage the disclosure of problems, whereas weaker or judgmental ties may deter it [21, 22]. Preferences for informal versus formal help sources (e.g., friends or family versus counsellors or GPs) are likely shaped not only by individual attitudes but also by the perceived availability and composition of one’s network [23]. Some evidence suggests that individuals with networks comprising diverse and longstanding relationships are more likely to draw on informal help [24, 25]; however, few studies explicitly distinguish between intentions to seek informal versus formal help in student populations. This limits our understanding of how structural and perceptual features interact to shape students’ support trajectories – a key concern for both theoretical and applied perspectives.

### Study objectives

This study aimed to map the structural characteristics of students’ personal support networks and examine how both structure and perceived support availability relate to mental health and help-seeking among UK undergraduates. Although theoretical models, such as SNT, SCT, and SBM, emphasise the importance of both, few studies have analysed these dimensions side by side within the same undergraduate sample, particularly in the context of UK higher education. We focused on four key network features – size, average relationship duration, relationship diversity, and support diversity – and tested whether these structural aspects predicted perceived support, and whether both structure and perception were independently associated with symptoms of depression and anxiety.

We made four key predictions. First (H1), that longer relationship duration, greater relationship diversity, and greater support diversity will be associated with higher perceived social support. Second (H2), higher perceived social support will predict lower levels of depression and anxiety, independent of network characteristics. Third (H3), higher perceived social support will indicate a greater intention to seek informal help, and a lower intention to seek formal help. Finally (H4), larger network size, longer relationship duration, greater relationship diversity, and greater support diversity will predict greater intention to seek informal help.

## Methods

### Participants and recruitment

This cross-sectional survey study was conducted between October 2023 and October 2024 using an online questionnaire hosted on Qualtrics. Participants were recruited via multiple channels, including social media (X/Twitter, Facebook, Instagram), physical flyers on a large UK university campus, emails circulated through university research networks, and online research participant platforms (SurveyCircle, WeParticipated). Social media posts were made once a month using the first author’s professional accounts and included a public digital flyer and accompanying text that detailed inclusion criteria, study description, and a direct survey link. No incentives were offered for participation.

Eligible participants were undergraduate students aged 18 or over, enrolled at a UK higher education institution, with sufficient English proficiency to complete the survey unaided. Participants were required to provide digital informed consent, pass a CAPTCHA bot-screening check (Qualtrics score ≥ 0.5) and complete 100% of survey items within 14 days. Postgraduate students, those studying outside the UK, those with limited English proficiency or those who did not consent were excluded. Responses identified as automated (Qualtrics score < 0.5) or incomplete after 14 days were also excluded. A total of 26 responses were removed on this basis (17 failed the CAPTCHA bot-check; 9 were incomplete), resulting in a final analytic sample of *N* = 287 UK-based undergraduate students.

Prior to data collection, an a priori power analysis was conducted using G*Power 3.1 [26]. This analysis indicated that a sample of approximately 118 participants would be required to detect a medium effect size (*f*^*2*^ = 0.15) [27], in a multiple regression model with up to 10 predictors, an alpha level of 0.05, and power (1 - *β*) of 0.80. All hypothesis-testing models were sufficiently powered. The most demanding model (formal help-seeking) had a small observed effect size (adjusted *R*² = 0.045, *f*² = 0.057). Post hoc power analysis indicated power = 0.90 with α = 0.05 and 10 predictors (*N* = 260), exceeding the conventional threshold of 0.80.

### Procedure

Participants accessed the survey via a secure Qualtrics link and provided digital informed consent before accessing the remainder of the survey. The survey was administered in a fixed order for all participants. First, the Perceived Support Network Inventory (PSNI) [28] based support network measure was completed, followed by the Multidimensional Scale of Perceived Social Support (MSPSS) [29], PHQ-9 [30], GAD-7 [31], and General Help-Seeking Questionnaire (GHSQ) [32]. Demographic questions (age bracket, gender identity, sexual orientation, ethnicity, university and degree of study, first-generation university status, and international student status) were presented at the end. The survey took an average of 15–20 min to complete. Participants were informed that they could save their response and return to it later within the 14-day window. Upon completion, participants were debriefed and provided with information about mental health support resources.

### Measures

#### Support network characteristics

 We assessed personal support network structure using a shortened version of the PSNI [28]. In this version, we removed the original items on reciprocity and conflict to align the PSNI with our conceptual focus on structural features of support networks. This allowed us to repurpose the measure as a structural mapping tool rather than a hybrid structural-perceptual index, ensuring conceptual consistency across analyses and reducing cognitive load for participants.

Participants were instructed: “Write the initials of all the people you would go to if you needed support or help during a stressful time in your life. You do not have to fill out this list in any particular order and you do not have to use all of the spaces available. You will be able to include a maximum of 15 people.” For each person listed, participants then reported: [1] the type of relationship (e.g., friend, parent, partner) [2], the approximate duration of that relationship in years, and [3] the types of support provided by that person, chosen from six categories (emotional, financial, instrumental, informational, appraisal, and social participatory support). These data were used to map each participant’s support network size, composition, and range of support functions.

From the PSNI data, we derived four indices to summarise each participant’s support network structure: network size, average relationship duration, relationship diversity, and support diversity. Network size was defined as the number of individuals listed in one’s support network (range 0–15). Average relationship duration was calculated as the mean length (in years) of all reported relationships in the network. Relationship diversity was calculated by summing the number of distinct relationship types represented in each participant’s network (range 0–9) [24]. For example, a participant who listed three friends, two parents, and one lecturer would have a relationship diversity of 3. Support diversity was calculated by summing the number of distinct types of support endorsed across each participant’s entire network, based on participants’ selections for each name individual (range 0–6). Higher diversity indices indicated a broader variety of relationship types or support functions, respectively.

The original PSNI has demonstrated acceptable internal consistency for its support indices (α = 0.76) [28], but its structural mapping outputs have not yet undergone psychometric validation in the same way. In this study, we treated the PSNI primarily as a structured tool for generating ego network characteristics. While this approach aligns conceptually with our network-focused aims, internal validity may be reduced due to the adaptation of the tool from its original form.

#### Perceived social support

The perceived availability of support was measured using the MSPSS [29]. The MSPSS is a 12-item questionnaire that evaluates how an individual perceives support is available from friends, family, and a significant other, as well as overall perceptions of support availability across these sources. Items are rated on a 7-point Likert scale (1 = Very strongly disagree to 7 = Very strongly agree). Perceived social support was scored as the average of all 12 items, yield an overall score from 1 to 7. We also computed MSPSS subscale scores (4 items each) for perceived support from significant other(s), family, and friends, though our analyses focused on the overall score. The MSPSS has demonstrated high internal consistency in student samples (α = 0.85–0.91) [33].

#### Depression and anxiety symptoms

 Symptoms of depression were measured with the 9-item PHQ-9 [30], and symptoms of anxiety were measured with the 7-item GAD-7 [31]. Each instrument asks how often in the past two weeks the respondent has been bothered by various problems (i.e., “little interest or pleasure in doing things” for depression; “feeling anxious, nervous or on edge” for anxiety). Items are rated on a 4-point scale from 0 (not at all) to 3 (nearly every day). Depression and anxiety scores were calculated as the sum of the PHQ-9 and GAD-7 item scores, respectively, with higher totals indicating more severe symptoms. PHQ-9 total scores range from 0 to 27 and GAD-7 scores range from 0 to 21.

Both the PHQ-9 and GAD-7 are well-validated; the PHQ-9 has shown good internal consistency in student populations (α > 0.80) [30], and the GAD-7 has similarly strong reliability in both clinical and non-clinical samples (α > 0.85) [31].

#### Help-seeking intentions

 Intentions to seek help were assessed using the GHSQ [32], adapted to include university-specific sources of help, such as flatmates, lecturers/seminar leaders, academic advisors/tutors, and non-academic university staff. Participants rated how likely they would be to seek help from each of 12 potential sources if they were experiencing: (a) personal or emotional problems, or (b) suicidal thoughts. Each source (partner, friends, parent, other family member, flat/housemate, mental health professional, helpline, GP/doctor, lecturer/seminar lead, academic advisor/tutor, non-academic university staff, and religious leader) was rated on a 7-point likelihood scale (1 = Extremely unlikely to 7 = Extremely likely). Higher scores indicate a greater likelihood of seeking help.

Help-seeking scores were calculated following standard scoring procedures [32], producing three distinct indices: overall help-seeking likelihood, informal help-seeking likelihood, and formal help-seeking likelihood. We averaged responses across the two scenarios to yield an overall help-seeking intention score. We then calculated two subscale indices based on source types: and informal help-seeking score (the average likelihood of seeking help from friends, partner, parents, other family, flat/housemates) and a formal-help seeking score (the average likelihood of seeking help from mental health professional, helpline, GP/doctor, lecturer/seminar lead, academic advisor/tutor, non-academic university staff, or religious leader). Scores ranged from 0 to 7, with higher scores indicating a greater likelihood of seeking help. The GHSQ has shown good internal consistency in university student samples (α = 0.85–0.90) [32].

#### Exploratory demographic predictors

Participants reported their gender identity, sexual orientation, ethnicity, first-generation university status, and international student status. These variables were included as exploratory predictors in the regression analyses. Participants who chose not to disclose their ethnicity (*n* = 6) or who identified as non-binary (*n* = 4) were excluded from regression models due to small group sizes that precluded meaningful comparisons.

For regression analyses, all categorical demographic variables were dummy coded. Gender was coded as 0 = male (reference), 1 = female; sexual orientation as 0 = heterosexual (reference), 1 = non-heterosexual; ethnicity as 0 = White (reference), 1 = Minority ethnic background; first-generation status as 0 = continuing-generation student (reference), 1 = first-generation student; and international student status as 0 = home student (reference), 1 = international student.

### Data cleaning and preparation

All continuous variables were assessed for normality using histograms and Shapiro–Wilk tests. Several variables—including network size, average relationship duration, relationship diversity, and perceived support—showed non-normal distributions, so Spearman’s rank-order correlations were used for bivariate analyses. Multicollinearity was assessed using Pearson’s correlations and variance inflation factors (VIF). Due to high intercorrelations among the MSPSS subscales (*r* >.80), only the total perceived support score was retained to avoid redundancy.

For each multiple regression model (predicting depression, anxiety, informal help-seeking, and formal help-seeking), potentially influential outliers were identified using a combination of standardised residuals (|z| >3.00), Mahalanobis distance, leverage values, and Cook’s distance. Cases exceeding two or more thresholds were flagged based on stringent criteria (*p* <.001). Each model was then run with and without flagged cases. Although effect sizes (standardised beta coefficients) and significance levels remained largely consistent, exclusion of outliers slightly improved adherence to regression assumptions, particularly normality and homoscedasticity of residuals. Residuals were approximately normally distributed (as confirmed by histograms and P–P plots), with no evidence of heteroscedasticity or non-linearity. Tolerance values were all > 0.40, indicating no multicollinearity.

Given these improvements and minimal interpretive differences, primary results are reported based on models with outliers excluded. Full sensitivity analyses using the full sample are presented in Supplementary Tables S1–S4. Final sample sizes for the reported models were: depression (*N* = 261), anxiety (*N* = 266), informal help-seeking (*N* = 260), and formal help-seeking (*N* = 254), all exceeding recommended minimums for multiple regression (15 cases per predictor). As this was an exploratory analysis of pre-specified models, no corrections were applied for multiple comparisons; results should therefore be interpreted in light of this.

### Data analysis

All analyses were conducted in SPSS v29 [34]. Descriptive statistics were computed to summarise the sample’s demographic characteristics and key study variables (support network indices, perceived support, mental health scores, and help-seeking scores). To test H1, we examined associations between support network structure and perceived social support using Spearman’s rank-order correlations. To test H2, we conducted multiple linear regression analyses predicting depression and anxiety. To test H3 and H4, we conducted two multiple regressions predicting the likelihood of seeking informal help and the likelihood of seeking formal help, respectively. All regression models included the four network structure indices (network size, average relationship duration, relationship diversity, support diversity) and the overall perceived support score as key predictors. We also entered five demographic covariates (gender, sexuality, ethnicity, first-generation status, and international student status) as exploratory predictors. We report unstandardised coefficients (*B*) and standardised coefficients (β) for all regression models, and statistical significance was set at *p* <.05 (two-tailed) for all hypothesis tests.

## Results

### Sample characteristics

The final sample included 287 undergraduate students attending 68 UK universities, spanning England, Scotland, Wales, and Northern Ireland. The majority identified as female (64.8%), White (61.7%), and heterosexual (81.9%). Additionally, 42.5% were first-generation students and 47.4% were international students. Table 1 presents full demographic information.

**Table 1:** Full sample characteristics (*N* = 287)

Characteristic	*n*	%
Age		
18–21 years	102	35.5
22–25 years	112	39.0
Over 25 years	73	25.4
Gender		
Female	186	64.8
Male	97	33.8
Non-binary	4	1.4
Sexual Orientation		
Heterosexual	235	81.9
Non-heterosexual	52	18.1
Ethnicity		
White	177	61.7
Asian	57	19.9
Black	20	7.0
Mixed/Multiple ethnic background	17	5.9
Arab	5	1.7
Other/unspecific	5	1.7
Prefer not to say	6	2.1
Enrolment status		
Full-time	247	86.1
Part-time	40	13.9
First-generation university student		
Yes	122	42.5
No	165	57.5
International student		
Yes	136	47.4
No	151	52.6
Reported mental health condition		
Yes	76	26.5
No	211	73.5

### Descriptive statistics for key variables

On average, students reported moderately sized personal networks (*M* = 4.02 members, *SD* = 3.15), with relationships lasting approximately 12 years (*M* = 11.97, *SD* = 8.84). Relationship and support diversity scores indicated that participants typically relied on multiple types of connections and support functions. Perceived social support was high, especially from significant others, though support from family and friends was also strong. Mean depression (PHQ-9) and anxiety (GAD-7) scores were in the mild range but spanned the full possible range. Students reported moderate intentions to seek help from informal sources and slightly lower intentions for formal sources, with wide variability across the sample. Table 2 summarises the key study variables in full.

**Table 2:** Descriptive statistics for main study variables (*N* = 287)

Variable	M	SD	Observed Range
Network characteristics (PSNI)			
Network size (number of members)	4.02	3.15	0–15
Relationship duration (years)	11.97	8.84	0–43
Relationship diversity (distinct relationship types)	2.24	1.20	0–5
Support diversity (support types available across network)	4.80	1.90	0–6
Perceived social support (MSPSS)			
Overall perceived support	5.02	1.15	1.25-7
Significant other support	5.16	1.52	1–7
Friend support	4.97	1.28	1–7
Family support	4.94	1.34	1–7
Mental health symptoms			
Depression (PHQ-9)	9.96	4.65	0–24
Anxiety (GAD-7)	8.68	3.92	0–21
Help-seeking intentions (GHSQ)			
Likelihood of overall help-seeking	3.01	0.72	1.25–5.58
Likelihood of informal help-seeking	3.64	0.93	0.80–6.10
Likelihood of formal help-seeking	2.56	0.90	0.29–5.79

### H1: associations between network structure and perceived support

To test Hypothesis 1, we examined Spearman’s correlations between structural network features and perceived social support scores. All four structural indicators – network size, relationship duration, relationship diversity, and support diversity – were positively associated with perceived support, both overall and across subscales. Among these, network size and both diversity indices were most strongly correlated with family support, though all associations were moderate and interpretation should be cautious given the lack of formal tests comparing correlation strength.

Overall, the strength of these associations, though moderate, suggest that students with broader, more varied, and more established networks tend to perceive greater support from those around them. All correlations were statistically significant at *p* <.001. See Table 3 for the full correlation matrix.

**Table 3:** Spearman’s correlations between support network characteristics and perceived social support (*N* = 287)

Network characteristic	Perceived social support			
	Partner support	Family support	Friend support	Overall support
Network size	0.21	0.26	0.22	0.27
Mean relationship duration	0.17	0.24	0.19	0.23
Relationship diversity	0.25	0.27	0.17	0.26
Support diversity	0.20	0.27	0.18	0.25

All correlations are significant at the *p* <.001

### H2: associations between support and mental health symptoms

To test Hypothesis 2, we conducted two multiple linear regressions predicting symptoms of depression and anxiety from perceived social support, structural network characteristics, and exploratory demographics. The model predicting depression explained 7% of the variance (adjusted *R*^2^ = 0.067), while the model for anxiety explained 9% of the variance (adjusted *R*^2^ = 0.089).

Perceived support emerged as the strongest and most consistent predictor across both models. Controlling for network structure and demographic characteristics, students who felt more supported reported lower symptoms of depression (β=>−0.35, *p* <.001) and anxiety (β = >−0.38, *p* <.001). In practical terms, each 1-point increase in perceived support was associated with a 1.30-point decrease in depression scores (0–27 scale) and a 1.19-point decrease in anxiety scores (0–21 scale). While modest, these effects are meaningful at the population level and suggest that perceived support plays a critical role in protecting student mental health.

Network size was also significantly associated with symptom severity, but in the opposite direction. Students with larger support networks reported slightly higher symptoms of both depression (β = 0.26, *p* =.002) and anxiety (β = 0.19, *p* =.021). Each additional person in a student’s support network was associated with a 0.37-point increase in depression scores and a 0.25-point increase in anxiety scores. Though unexpected, these associations were small and may reflect strain from maintaining larger networks or unmet expectations within them.

Sexual orientation also predicted depression scores: non-heterosexual participants reported significantly more severe symptoms than heterosexual peers (β = 0.14, *p* =.017). No other demographic variables were significantly associated with depression or anxiety.

Ultimately, these findings support Hypothesis 2, demonstrating that perceived support independently predicts better mental health outcomes, even after accounting for demographic background and the structure of students’ personal networks.

Full model statistics are reported in Tables 4 and 5. Sensitivity analyses including outliers produced substantively similar results and are presented in Supplementary Table S1-2.

**Table 4:** Multiple linear regression predicting depression scores (*N* = 253)

Predictor	B	β	95% CI	*p*
(Constant)	15.03		[11.88, 18.17]	< 0.001
Female (ref = male)	−0.16	−0.02	−1.16, 0.84]	0.756
Non-heterosexual (ref = heterosexual)	1.55	0.14	[0.28, 2.82]	0.017
Minority ethnic background (ref = white)	−0.38	−0.05	−1.35, 0.60]	0.447
First-generation student	−0.31	−0.04	−1.26, 0.65]	0.527
International student	−0.52	−0.07	−1.49, 0.45]	0.290
Network size	0.37	0.25	[0.13, 0.60]	0.002
Mean relationship duration	0.06	0.13	>−0.00, 0.12]	0.053
Relationship diversity	−0.33	−0.10	−0.94, 0.27]	0.276
Support diversity	0.23	0.11	−0.12, 0.58]	0.203
Overall perceived support	−1.30	−0.35	−1.82,−0.79	< 0.001

*B* unstandardised beta, *β* standardised beta

**Table 5:** Multiple linear regression predicting anxiety scores (*N* = 258)

Predictor	B	β	95% CI	*p*
(Constant)	13.36		[10.66, 16.07]	< 0.001
Female (ref = male)	−0.17	−0.02	−1.03, 0.69]	0.692
Non-heterosexual (ref = heterosexual)	−0.39	−0.04	−1.46, 0.68]	0.476
Minority ethnic background (ref = white)	−0.04	−0.01	−0.88, 0.81]	0.929
First-generation student	−0.10	−0.02	−0.93, 0.72]	0.804
International student	−0.09	−0.01	−0.92, 0.75]	0.843
Network size	0.25	0.19	[0.04, 0.46]	0.021
Mean relationship duration	0.04	0.01	−0.01, 0.09]	0.146
Relationship diversity	−0.31	−0.10	−0.83, 0.22]	0.249
Support diversity	0.18	0.10	−0.12, 0.49]	0.242
Overall perceived support	−1.19	−0.38	−1.63,−0.76	< 0.001

*B* unstandardised beta, *β* standardised beta

### H3/H4: predicting help-seeking intentions from perceived and structural support

To test Hypotheses 3 and 4, we rain two multiple linear regressions predicting informal and formal help-seeking intentions from perceived support, structural network characteristics, and exploratory demographic variables. The model predicting informal help-seeking explained 15% of the variance (adjusted *R*^2^ = 0.146), while the model for formal help-seeking explained just over 4% (adjusted *R*^2^ = 0.045).

As predicted in H3, perceived social support showed opposing associations with help-seeking direction. Students who felt more supported were significantly more likely to seek help from informal sources (β = 0.23, *p* <.001), but less likely to seek help from formal sources (β = −0.15, *p* =.002), even after controlling for network structure and demographics. In practical terms, a 1-point increase in perceived support (on a scale of 1–7) was linked to a 0.23-point increase in students’ likelihood of seeking help from informal sources (e.g., friends, family) and a 0.15-point decrease in their likelihood of turning to formal services (e.g., GP, university staff), both measured on a 1–5 scale. While these shifts may seem small at the individual level, they reflect meaningful trends across the sample, suggesting that stronger perceptions of support can increase reliance on trusted personal networks while reducing demand for professional help.

Contrary to H4, only one structural characteristic – support diversity – significantly predicted informal help-seeking intentions, and in the opposite direction to that hypothesised. Students whose networks offered a wider range of support types were less likely to seek help from informal sources (β = −0.32, *p* <.001). Each additional type of support available across the network (range: 0–6) was associated with a 0.14-point decrease in informal help-seeking intentions (range: 1–5). This finding suggests that even when students have access to varied forms of support within their personal networks, they may not feel inclined to approach friends or family when facing mental health concerns. No other structural variables were significantly associated with informal help-seeking. As such, these results do not support H4 and indicate that greater structural provision of support does not necessarily translate into greater willingness to seek help informally.

Full model statistics are reported in Tables 7 and 6. Sensitivity analyses including outliers produced substantively similar results and are presented in Supplementary Table S3-4.

**Table 6:** Multiple linear regression predicting informal help-seeking (*N* = 260)

Predictor	B	β	95% CI	*p*
(Constant)	3.42		[2.76, 4.07]	< 0.001
Female (ref = male)	−0.37	−0.21	−0.58,−0.16	< 0.001
Non-heterosexual (ref = heterosexual)	0.25	0.11	−0.01, 0.51]	0.062
Minority ethnic background (ref = white)	0.31	0.18	[0.10, 0.51]	0.003
First-generation student	−0.19	−0.11	−0.38, 0.01]	0.065
International student	−0.04	−0.02	−0.24, 0.16]	0.725
Network size	0.01	0.03	−0.04, 0.06]	0.734
Mean relationship duration	−0.00	−0.04	−0.02, 0.01]	0.592
Relationship diversity	0.08	0.12	−0.04, 0.20]	0.186
Support diversity	−0.14	−0.32	−0.21,0.07	< 0.001
Overall perceived support	0.23	0.29	[0.12, 0.34]	< 0.001

*B* unstandardised beta, β standardised beta

**Table 7:** Multiple linear regression predicting formal help-seeking (*N* = 254)

Predictor	B	β	95% CI	*p*
(Constant)	3.38		[2.77, 3.99]	< 0.001
Female (ref = male)	0.04	0.02	−0.16, 0.23]	0.718
Non-heterosexual (ref = heterosexual)	0.16	0.08	−0.08, 0.39]	0.193
Minority ethnic background (ref = white)	0.12	0.08	−0.06, 0.31]	0.196
First-generation student	−0.17	−0.11	−0.35, 0.02]	0.074
International student	−0.12	−0.08	−0.30, 0.07]	0.216
Network size	0.02	0.08	−0.03 0.07]	0.360
Mean relationship duration	−0.00	−0.05	−0.02, 0.01]	0.504
Relationship diversity	−0.03	−0.04	−0.14, 0.09]	0.648
Support diversity	0.05	0.13	−0.02, 0.12]	0.148
Overall perceived support	−0.15	−0.23	−0.25, −0.05	0.002

*B *unstandardised beta, *β* standardised beta

## Discussion

This study examined the relationship between the structural features and perceived availability of UK-based undergraduates’ personal support networks and their mental health and help-seeking outcomes. Consistent with theoretical frameworks that distinguish between support availability and support structure (16, 35) perceived social support emerged as the most consistent predictor across all outcome measures. Students who perceived a high availability of support reported significantly less severe depression and anxiety symptoms, were more likely to seek help from informal sources, and were less likely to seek help from formal sources. By contrast, objective network characteristics showed more modest and inconsistent associations with outcomes: having a larger network was associated with higher perceived support but also predicted more severe symptoms of depression and anxiety. Moreover, contrary to our predictions, greater support diversity was linked to a lower likelihood of informal help-seeking. Overall, these results align with our theoretically grounded approach, which integrates Social Network theory (SNT), Social Capital theory (SCT), and the Stress-Buffering Model (SBM) to explain both structural and perceptual dynamics of support. While SNT and SCT highlight how network configuration affects access to resources [7, 8], the SBM underscores the protective psychological impact of perceiving support to be available [16] – providing a strong interpretative lens for the observed results.

### Perceived support and network characteristics

Hypothesis 1 was fully supported: all four network characteristics that were measured (size, average relationship duration, relationship diversity, support diversity) showed positive associations with perceived support availability. These findings are consistent with prior research suggesting that individuals with larger and more varied support networks are more likely to believe that help will be available when needed [11, 12]. In particular, longer-standing relationships may foster trust and perceived dependability, while diverse relationship types likely signal access to a broader range of support functions.

The relevance of these associations may be amplified in a UK undergraduate context, where support networks are frequently disrupted or reconstructed during the transition to university life [10]. Many students relocate for university, leaving behind established support systems and needing to rapidly rebuild new ones across campus, digital, and home-based settings. The positive associations between perceived support and structural features may therefore reflect not just volume or variety, but a student’s ability to maintain relational continuity or re-establish diverse networks in a demanding social context. In this sense, perceived support may serve as a psychological proxy for network resilience: students who can preserve or adapt their networks amid disruption may also retain greater confidence in their ability to access support.

It is also possible that in the UK context, where public discourse around mental health is increasingly visible but student services remain under-resourced [35], students may lean more heavily into informal support, making the structure of their network particularly salient to their perceptions of support availability [36]. While the MSPSS does not capture enacted support or support satisfaction, it does tap into students’ confidence in the social scaffolding surrounding them. Our findings suggest that the structure of that scaffolding – its breadth, diversity, and stability – continues to matter, even in a cultural setting where peer-based support is often framed as effective but inconsistently accessible [5].

Overall, these findings underscore the importance of seeing support networks not only as passive background structures but as active contexts that shape how students evaluate their access to support. Structural features do not guarantee support, but they do appear to create the conditions in which support is perceived to be present and available.

### Support networks, perceived support and mental health

Hypothesis 2 was fully supported: higher perceived social support predicted lower levels of depression and anxiety, even after accounting for structural network characteristics. This reinforces a substantial body of evidence identifying perceived support as one of the most robust and consistent protective factors for psychological wellbeing, particularly among young adults and students [27, 37, 38]. Crucially, this effect was independent of how large or structurally diverse a student’s network was. The belief that support is available, regardless of how many people are in a student’s personal network, appears central to psychological resilience [17].

Importantly, although some effect sizes were small, their practical significance may still be meaningful in a student population where even most improvements in mental health indicators can translate to better academic engagement, retention, and quality of life [37]. For example, a one-point increase in perceived support was associated with a measurable reduction in depression and anxiety symptoms, aligning with past work showing even small support-related shifts can reduce the onset or escalation of clinical distress [27].

These results support models that conceptualise perceived support as a cognitive-affective buffer that helps individuals manage stress and uncertainty [39]. The expectation that others will provide care when needed may reduce anticipatory anxiety, reinforce a sense of belonging, and enhance self-efficacy in coping with stressors [40]. In the UK university context, where formal mental health services remain overstretched and often difficult to access [41, 42], this internalised sense of support may play a vital role in mitigating psychological distress.

Interestingly, when perceived support was held constant, students with larger networks reported more severe symptoms of depression and anxiety. While this may seem counterintuitive, similar patterns have been observed in other research, suggesting that larger networks can introduce social strain or emotional fatigue, particularly when relationships lack reciprocity or stability [16, 43]. For students, managing a vast network may carry hidden social costs – more obligations, greater relational complexity, and the potential for interpersonal conflict – all of which can compound stress and worsen mental health. Alternatively, students experiencing psychological distress may attempt to expand their networks in search of relief, pointing to the possibility of reverse causality. Given the cross-sectional nature of our data, causality cannot be determined, but these findings challenge the assumption that larger networks are inherently protective.

None of the other structural variables – relationship duration, relationship diversity, or support diversity – significantly predicted depression or anxiety when perceived support was controlled for. This adds to growing evidence that it is not the objective makeup of a support network, but rather a subjective sense of support availability, that most directly influences psychological outcomes [17, 27] Recognising this distinction shifts the focus from counting social connections to understanding how support is internalised and experienced.

### Support networks, perceived support and help-seeking

Hypothesis 3 was fully supported: higher perceived social support was associated with greater intention to seek help from informal sources and lower intention to seek help from formal sources. This suggests that when students feel confident in the availability of interpersonal support, they are more likely to manage emotional difficulties within their personal networks. Prior research has shown that strong perceived support can reduce reliance on professional services, either due to informal relationships providing sufficient help or due to young people feeling less urgency to seek external input [21, 44].

The findings for Hypothesis 3 also have practical relevance: although structural predictors showed small or null effects, perceived support was consistently linked with students’ intention to seek informal help and avoid formal services. This suggests that students may draw on trusted relationships before escalating to professional care. This is consistent with prior findings from Boldero and Fallon [45], Wilson and colleagues [32], and Gulliver and colleagues [22], which show that perceived emotional proximity and social norms heavily shape early-stage help-seeking behaviour in young adults.

This dynamic, however, is complex. Informal support can offer timely, low-barrier assistance, but it also may delay engagement with professional help when such support is needed. Our findings mirror this dual pattern, highlighting how perceived support simultaneously facilitates informal help-seeking and may displace formal service use – a tension documented in both student and general populations [22, 46].

In contrast, Hypothesis 4 was not supported. None of the structural characteristics – size, relationship duration, relationship diversity, support diversity – positively predicted students’ intention to seek informal help. This contradicts our initial predictions that having a broader or more diverse network would increase informal help-seeking behaviour. Support diversity showed a small but significant negative association with informal help-seeking, directly contradicting our prediction.

Although prior studies have suggested that larger or more diverse networks might facilitate informal help-seeking [46, 47], others have found these effects to be inconsistent or dependent on relationship quality and salience [5, 48]. One possible explanation is that students with such support diversity may experience functional fragmentation, where emotional trust is not concentrated in a single source. As Boldero and Fallon [45] and Thoits [49] argue, the presence of multiple support types does not guarantee emotional proximity or help-seeking comfort. Alternatively, students with high support diversity may be more used to managing their needs independently by drawing on different forms of help and may not conceptualise these actions as “help-seeking” in the traditional sense [21]. These interpretations remain speculative, but they highlight that access to a wide array of support types does not necessarily translate into a greater willingness to seek help, especially if those supports are experienced as functionally compartmentalised or lacking emotional depth. Moreover, even when support is structurally available, students may lack the confidence, skills, or knowledge needed to navigate these networks effectively, limiting their ability to access or activate the help they need.

Taken together, our findings highlight that students’ help-seeking preferences are shaped more by their perceptions of support than by the structural features of their networks. Perceived social support was the strongest and most consistent predictor across models, associated with a greater likelihood of seeking informal help and a lower likelihood of seeking formal help. In contrast, structural characteristics such as network size, relationship duration, and relationship diversity had no meaningful influence. The one exception—support diversity—was negatively associated with informal help-seeking, suggesting that access to a broad range of support types may not necessarily encourage help-seeking if those supports are experienced as fragmented or impersonal. These results underscore a critical distinction: while structural network features may create the conditions for support, it is students’ confidence in the availability of that support that determines whether and how they reach out for help.

### Implications

These findings, combined with the understanding that perceptions of support are modifiable, underscore the importance of addressing students’ access to support as well as their belief in its availability and dependability. In UK universities, where students often navigate transitional networks and face pressures to self-manage their mental health, perceived support serves as a psychological mediator. Initiatives fostering relational trust and emotional safety – such as peer mentoring and small-group interventions – have shown positive outcomes in diverse student populations. For instance, peer mentoring programmes have been associated with improved mental health and wellbeing among university students [50], and peer-led support groups have demonstrated benefits in reducing anxiety and stress [5]. However, our results caution against assuming that larger networks inherently offer protection; quantity without quality may increase strain or mask unmet need. The current study supports the argument that structural network features may be necessary, but not sufficient for positive mental health outcomes; rather, students’ subjective experience of support – shaped by perceived availability – acts as the more proximal determinant of psychological wellbeing. As such, peer support initiatives, while capable of expanding social contacts, do not automatically foster deep, meaningful relationships; research with mental health peer support workers has highlighted that such relationships can often remain superficial or constrained by role boundaries [51]. Universities should, therefore, prioritise initiatives that support the development of sustained, emotionally meaningful peer relationships—such as longitudinal peer mentoring schemes, small-group belonging interventions, or facilitated discussion spaces—rather than relying solely on open-access peer-led support groups, which may risk fostering superficial connections. Rather than focusing solely on increasing support options, mental health services and policies might prioritise ways to strengthen perceived availability – such as embedding peer support, increasing staff continuity, or providing clearer communication about where and when help is available.

### Contribution to knowledge

This study extends the social support literature by focusing on UK undergraduates – a group often overlooked in favour of international or general adult populations navigating broader social contexts. While prior research has examined personal support networks in general population samples, where both structural features and support typically show strong protective associations with wellbeing [16], our findings suggest that in undergraduate students, perceived support plays a comparatively stronger role. In contrast, structural features exert less predictive influence. This indicates that support networks and social support may operate differently in student populations, where subjective perceptions appear to carry greater weight than objective network characteristics, compared to general adult samples, where both dimensions tend to align more closely in shaping mental health outcomes.

To our knowledge, it is among the first to examine both structural and perceived dimensions of support within the same student sample, offering new evidence that perceived support plays a stronger role than objective network features in predicting mental health and help-seeking. Importantly, we differentiated between informal and formal help-seeking, highlighting both the protective and potentially delaying role of strong personal support networks. Additionally, the unexpected negative association between support diversity and informal help-seeking challenges assumptions that having access to more support types is always beneficial, pointing to the need for more nuanced models of how students engage with their networks. Methodologically, our approach demonstrates the value of assessing structural and subjective support simultaneously to better understand their co-dependency and interplay.

### Strengths and limitations

This study offers a comprehensive analysis of both structural and perceived support in a large and diverse sample of UK undergraduates, using validated measures and detailed network data. By distinguishing between informal and formal help-seeking and including both objective and subjective support variables, the study provides a nuanced understanding of how support relates to student mental health and help-seeking.

Nonetheless, several limitations must be acknowledged. First, the cross-sectional design prevents causal inference; we cannot determine whether perceived support improves mental health or whether distress impairs perceptions of support availability. Second, the self-selecting nature of the sample and low number of participants per university may limit generalisability. Students more confident discussing mental health or engaged with support topics may have been overrepresented, potentially inflating associations. Third, the small number of non-binary participants meant we could not include them in regression analyses—an important gap given evidence that gender minorities may experience distinct support challenges.

Although the shortened PSNI enabled efficient data capture, capping the number of network members at 15 may have under-represented students with larger or more diffuse networks. This choice reduced participant burden but may have constrained our ability to fully capture network complexity. Finally, while we assessed network structure and perceived availability, we did not measure the quality or dynamics of support. Future research could include relational satisfaction or empathy to offer a more complete account of how support functions in student mental health and help-seeking.

### Future research directions

Future research should prioritise longitudinal designs to examine how students’ support networks and perceptions evolve over time, particularly during key transitions such as moving away from home, studying abroad, or graduating. Tracking changes in network structure and perceived support across the university journey would help clarify causal pathways – for instance, whether increased perceived support predicts improved mental health, or vice versa. In parallel, qualitative or mixed-methods approaches are needed to explore how students interpret, utilise, and evaluate different forms of support. Interviews or focus groups could highlight support preferences, help-seeking decisions, and the lived experience of navigating diverse or fragmented networks. Together, these approaches would deepen our understanding of how support operates in student life and inform more tailored, context-sensitive interventions.

## Conclusion

This study highlights the pivotal role of perceived support availability in shaping undergraduate mental health and help-seeking. While larger and more diverse support networks contributed to stronger perceptions of support, it was students’ belief in the availability of support, not network size or composition, that most strongly predicted decreased distress and greater informal help-seeking. These findings suggest that wellbeing initiatives should focus not only on expanding social opportunities, but on fostering emotionally secure, trusting relationships. Supporting students to build a small number of meaningful connections – and ensuring those with strong informal networks still feel comfortable accessing professional help – may be key to improving mental health outcomes.

## Supplementary Information

Supplementary Material 1.

Supplementary Material 2.

## Data Availability

The datasets analysed during the current study are available from the corresponding author on reasonable request.
