# Supplementary material for: Mapping support systems: a cross-sectional examination of personal support networks, perceived support, mental health outcomes, and help-seeking behaviours among UK undergraduate students
Source: BMC Public Health. 2025 Sep 30;25:3190. doi: 10.1186/s12889-025-24360-1 (PMC12487519; doi:10.1186/s12889-025-24360-1)
Supplement: Supplementary file 1 — Supplementary Material 1. [file 12889_2025_24360_MOESM1_ESM.docx]

**Qualtrics Survey**

Questionnaire measuring properties of support networks

The first section of this survey has been designed to collect information about your support network. We are collecting this information so we can build a picture of what the support networks of undergraduate students in the UK look like – e.g., size of network, length of relationships, types of relationships.

**Support Network List**

**Instructions:** Write the initials of all the people you would go to if you needed support or help during a stressful time in your life. Estimate the duration of your relationship to this person (e.g., the length of time you have known each other in years). From the dropdown list, pick the label that describes your relationship with each person or include a relationship label of your own. Finally, check all of the types of support this person provides you with. Definitions of the support types can be found below and will be visible while you complete this activity:

- Emotional support
  - They ensure you feel cared for and loved.
- Financial support
  - They provide money to enable you to meet your needs, such as to buy groceries or medication.
- Informational support
  - They provide you with knowledge or facts, such as advice, feedback on actions, or mentoring.
- Instrumental support
  - They are actively involved by taking responsibilities or providing acts of service, such as helping you do your laundry or cooking you a meal.
- Appraisal support
  - They provide information that is useful for self-evaluation, such as reminding you of the qualities that equip you to cope with stressful situations.
- Social participation
  - They help or encourage you to participate in your community or society.

You do not have to fill out this list in any particular order. You do not have to use all of the spaces available. You will be able to include a maximum of 15 people.

| Relationship Information | | | Support Types | | | | | |
| --- | --- | --- | --- | --- | --- | --- | --- | --- |
| Initials | Length of relationship (in years) | Relationship type* | Emotional | Financial | Informa-tional | Instrumental | Appraisal | Social partici-pation |
|  |  | ↓ | ☐ | ☐ | ☐ | ☐ | ☐ | ☐ |
|  |  | ↓ | ☐ | ☐ | ☐ | ☐ | ☐ | ☐ |
|  |  | ↓ | ☐ | ☐ | ☐ | ☐ | ☐ | ☐ |
|  |  | ↓ | ☐ | ☐ | ☐ | ☐ | ☐ | ☐ |
|  |  | ↓ | ☐ | ☐ | ☐ | ☐ | ☐ | ☐ |
|  |  | ↓ | ☐ | ☐ | ☐ | ☐ | ☐ | ☐ |
|  |  | ↓ | ☐ | ☐ | ☐ | ☐ | ☐ | ☐ |
|  |  | ↓ | ☐ | ☐ | ☐ | ☐ | ☐ | ☐ |
|  |  | ↓ | ☐ | ☐ | ☐ | ☐ | ☐ | ☐ |
|  |  | ↓ | ☐ | ☐ | ☐ | ☐ | ☐ | ☐ |
|  |  | ↓ | ☐ | ☐ | ☐ | ☐ | ☐ | ☐ |
|  |  | ↓ | ☐ | ☐ | ☐ | ☐ | ☐ | ☐ |
|  |  | ↓ | ☐ | ☐ | ☐ | ☐ | ☐ | ☐ |
|  |  | ↓ | ☐ | ☐ | ☐ | ☐ | ☐ | ☐ |
|  |  | ↓ | ☐ | ☐ | ☐ | ☐ | ☐ | ☐ |

* Drop-down responses include:

- Spouse or partner
- Friend
- Parent
- Other family member
- Colleague
- Flat or housemate
- Healthcare professional (e.g., GP, mental health practitioner)
- University staff (e.g., lecturer, tutor, support staff)
- Religious leader (e.g., Rabbi, Priest)
- Other, please specify: ____________

Questionnaire measuring perceived social support

The second section of this survey has been designed to collect information about how you perceive the social support that is available to you. We are collecting this information so we can understand what support is offered to you and by whom.

**Instructions:** We are interested in how you feel about the following statements. Read each statement carefully and indicate how you feel about each.

|  | Very Strongly Disagree | Strongly Disagree | Mildly Disagree | Neutral | Mildly Agree | Strongly Agree | Very Strongly Agree |
| --- | --- | --- | --- | --- | --- | --- | --- |
| There is a special person who is around when I am in need | ☐ | ☐ | ☐ | ☐ | ☐ | ☐ | ☐ |
| There is a special person with whom I can share joys and sorrows | ☐ | ☐ | ☐ | ☐ | ☐ | ☐ | ☐ |
| My family really tries to help me | ☐ | ☐ | ☐ | ☐ | ☐ | ☐ | ☐ |
| I get the emotional help & support I need from my family | ☐ | ☐ | ☐ | ☐ | ☐ | ☐ | ☐ |
| I have a special person who is a real source of comfort to me | ☐ | ☐ | ☐ | ☐ | ☐ | ☐ | ☐ |
| My friends really try to help me | ☐ | ☐ | ☐ | ☐ | ☐ | ☐ | ☐ |
| I can count on my friends when things go wrong | ☐ | ☐ | ☐ | ☐ | ☐ | ☐ | ☐ |
| I can talk about my problems with my family | ☐ | ☐ | ☐ | ☐ | ☐ | ☐ | ☐ |
| I have friends with whom I can share my joys and sorrows | ☐ | ☐ | ☐ | ☐ | ☐ | ☐ | ☐ |
| There is a special person in my life who cares about my feelings | ☐ | ☐ | ☐ | ☐ | ☐ | ☐ | ☐ |
| My family is willing to help me make decisions | ☐ | ☐ | ☐ | ☐ | ☐ | ☐ | ☐ |
| I can talk about my problems with my friends | ☐ | ☐ | ☐ | ☐ | ☐ | ☐ | ☐ |

Questionnaire measuring mental health

The third section of this survey has been designed to collect information about your mental health. We are collecting this information so we can examine whether the characteristics of your support network impact your mental health, or vice versa.

**Instructions:** Thinking about the last 2 weeks, indicate how often you have been bothered by any of the following problems by ticking the relevant column.

|  | Not at all | Several days | More than half the days | Nearly every day |
| --- | --- | --- | --- | --- |
| Little interest or pleasure in doing things | ☐ | ☐ | ☐ | ☐ |
| Feeling down, depressed, or hopeless | ☐ | ☐ | ☐ | ☐ |
| Trouble falling asleep, staying asleep, or sleeping too much | ☐ | ☐ | ☐ | ☐ |
| Feeling tired or having little energy | ☐ | ☐ | ☐ | ☐ |
| Poor appetite or overeating | ☐ | ☐ | ☐ | ☐ |
| Feeling bad about yourself – or that you are a failure or have let yourself or your family down | ☐ | ☐ | ☐ | ☐ |
| Trouble concentrating on things, such as reading the news or watching television | ☐ | ☐ | ☐ | ☐ |
| Moving or speaking so slowly that other people have noticed, or the opposite – being so fidgety or restless that you have been moving around a lot more than usual | ☐ | ☐ | ☐ | ☐ |
| Thoughts that you would be better off dead or of hurting yourself some way | ☐ | ☐ | ☐ | ☐ |

**Instructions:** Thinking about the last 2 weeks, indicate how often you have been bothered by any of the following problems by ticking the relevant column.

|  | Not at all | Several days | More than half the days | Nearly every day |
| --- | --- | --- | --- | --- |
| Feeling nervous, anxious, or on edge | ☐ | ☐ | ☐ | ☐ |
| Not being able to stop or control worrying | ☐ | ☐ | ☐ | ☐ |
| Worrying too much about different things | ☐ | ☐ | ☐ | ☐ |
| Trouble relaxing | ☐ | ☐ | ☐ | ☐ |
| Being so restless that it is hard to sit still | ☐ | ☐ | ☐ | ☐ |
| Becoming easily annoyed or irritable | ☐ | ☐ | ☐ | ☐ |
| Feeling afraid, as if something awful might happen | ☐ | ☐ | ☐ | ☐ |

Questionnaire measuring help-seeking

The fourth section of this survey has been designed to collect information about who you would seek help from if you were experiencing certain problems. We are collecting this information so we can examine whether your help-seeking styles are linked with your support network characteristics.

**Instructions:** If you were having a personal or emotional problem, how likely is it that would seek help from the following people?

Please indicate your response by ticking the column that best describes your intention to seek help from each help source that is listed.

|  | Most Unlikely | Very Unlikely | Unlikely | Neutral | Likely | Very Likely | Most Likely | | N/A |
| --- | --- | --- | --- | --- | --- | --- | --- | --- | --- |
| Intimate partner (e.g., girl/boyfriend, partner) | ☐ | ☐ | ☐ | ☐ | ☐ | ☐ | ☐ | | ☐ |
| Friend (not related to you) | ☐ | ☐ | ☐ | ☐ | ☐ | ☐ | ☐ | | ☐ |
| Parent | ☐ | ☐ | ☐ | ☐ | ☐ | ☐ | ☐ | | ☐ |
| Other family member | ☐ | ☐ | ☐ | ☐ | ☐ | ☐ | ☐ | | ☐ |
| Flat or housemate | ☐ | ☐ | ☐ | ☐ | ☐ | ☐ | ☐ | | ☐ |
| Mental health professional (e.g., psychologist, social worker) | ☐ | ☐ | ☐ | ☐ | ☐ | ☐ | ☐ | | ☐ |
| Phone helpline (e.g., Samaritans) | ☐ | ☐ | ☐ | ☐ | ☐ | ☐ | ☐ | | ☐ |
| Doctor / GP | ☐ | ☐ | ☐ | ☐ | ☐ | ☐ | ☐ | | ☐ |
| Lecturer / seminar lead | ☐ | ☐ | ☐ | ☐ | ☐ | ☐ | ☐ | | ☐ |
| Academic advisor / tutor | ☐ | ☐ | ☐ | ☐ | ☐ | ☐ | ☐ | | ☐ |
| Non-academic university staff | ☐ | ☐ | ☐ | ☐ | ☐ | ☐ | ☐ | | ☐ |
| Religious leader (e.g., Rabbi, Priest) | ☐ | ☐ | ☐ | ☐ | ☐ | ☐ | ☐ | | ☐ |
| I would not seek help from anyone | ☐ | ☐ | ☐ | ☐ | ☐ | ☐ | ☐ | ☐ | |
| I would seek help from someone else (please specify): ------- | ☐ | ☐ | ☐ | ☐ | ☐ | ☐ | ☐ | ☐ | |

**Instructions:** If you were experiencing suicidal thoughts, how likely is it that would seek help from the following people?

Please indicate your response by ticking the column that best describes your intention to seek help from each help source that is listed.

|  | Most Unlikely | Very Unlikely | Unlikely | Neutral | Likely | Very Likely | Most Likely | | N/A |
| --- | --- | --- | --- | --- | --- | --- | --- | --- | --- |
| Intimate partner (e.g., girl/boyfriend, partner) | ☐ | ☐ | ☐ | ☐ | ☐ | ☐ | ☐ | | ☐ |
| Friend (not related to you) | ☐ | ☐ | ☐ | ☐ | ☐ | ☐ | ☐ | | ☐ |
| Parent | ☐ | ☐ | ☐ | ☐ | ☐ | ☐ | ☐ | | ☐ |
| Other family member | ☐ | ☐ | ☐ | ☐ | ☐ | ☐ | ☐ | | ☐ |
| Flat or housemate | ☐ | ☐ | ☐ | ☐ | ☐ | ☐ | ☐ | | ☐ |
| Mental health professional (e.g., psychologist, social worker) | ☐ | ☐ | ☐ | ☐ | ☐ | ☐ | ☐ | | ☐ |
| Phone helpline (e.g., Samaritans) | ☐ | ☐ | ☐ | ☐ | ☐ | ☐ | ☐ | | ☐ |
| Doctor / GP | ☐ | ☐ | ☐ | ☐ | ☐ | ☐ | ☐ | | ☐ |
| Lecturer / seminar lead | ☐ | ☐ | ☐ | ☐ | ☐ | ☐ | ☐ | | ☐ |
| Academic advisor / tutor | ☐ | ☐ | ☐ | ☐ | ☐ | ☐ | ☐ | | ☐ |
| Non-academic university staff | ☐ | ☐ | ☐ | ☐ | ☐ | ☐ | ☐ | | ☐ |
| Religious leader (e.g., Rabbi, Priest) | ☐ | ☐ | ☐ | ☐ | ☐ | ☐ | ☐ | | ☐ |
| I would not seek help from anyone | ☐ | ☐ | ☐ | ☐ | ☐ | ☐ | ☐ | ☐ | |
| I would seek help from someone else (please specify): ------- | ☐ | ☐ | ☐ | ☐ | ☐ | ☐ | ☐ | ☐ | |

Demographic questionnaire

The final section of this survey has been designed to collect some demographic information from you.

1. What is your age?
   1. ☐ 18-21
   2. ☐ 22-25
   3. ☐ 25+
2. What is your gender?
   1. ☐ Male
   2. ☐ Female
   3. ☐ Non-binary
   4. ☐ Other (please specify):
      1. ___________________
   5. Prefer not to say
3. Is your gender the same as the gender you were assigned at birth?
   1. ☐ Yes
   2. ☐ No
   3. ☐ Prefer not to say
4. What is your sexuality?
   1. ☐ Heterosexual / straight
   2. ☐ Homosexual / gay / lesbian
   3. ☐ Bisexual
   4. ☐ Other (please specify):
      1. ___________________
   5. Prefer not to say
5. What is your ethnic background?
   1. Asian / Asian British
      1. ☐ Indian
      2. ☐ Pakistani
      3. ☐ Bangladeshi
      4. ☐ Chinese
      5. ☐ Any other Asian background
   2. Black / Black British / Caribbean / African
      1. ☐ Caribbean
      2. ☐ African
      3. ☐ Any other Black, Black British, Caribbean, or African background
   3. Mixed / Multiple ethnic groups
      1. ☐ White and Black Caribbean
      2. ☐ White and Black African
      3. ☐ White and Asian
      4. ☐ Any other mixed or multiple ethnic backgrounds
   4. White
      1. ☐ English / Welsh / Scottish / Northern Irish / British
      2. ☐ Irish
      3. ☐ Gypsy / Irish Traveller
      4. ☐ Roma
      5. ☐ Any other White background
   5. Other ethnic groups
      1. ☐ Arab
      2. ☐ Any other ethnic group (please specify): ___________
6. What type of student are you?
   1. ☐ Full-time
   2. ☐ Part-time
7. What year of university are you in?
   1. ☐ 0 / Foundation year
   2. ☐ 1
   3. ☐ 2
   4. ☐ 3
   5. ☐ 4
   6. ☐ 5
   7. ☐ 6+
8. What university are you studying at?
   1. [drop down list]
9. What subject are you studying?
   1. [drop down list]
10. Are you the first person in your immediate family to go to university?
    1. ☐ Yes
    2. ☐ No
    3. ☐ Prefer not to say
11. Are you an international student – i.e., not from the UK?
    1. ☐ Yes
    2. ☐ No
    3. ☐ Prefer not to say
12. Have you been diagnosed with a mental health condition?
    1. ☐ Yes, but I would not like to specify
    2. ☐ Yes, and I would like to specify:
       1. ☐ Anxiety disorder(s)
       2. ☐ Bipolar disorder
       3. ☐ Body dysmorphic disorder
       4. ☐ Borderline / emotionally unstable personality disorder
       5. ☐ Depression
       6. ☐ Dissociation / dissociative disorder(s)
       7. ☐ Eating problems / eating disorder(s)
       8. ☐ Obsessive-compulsive disorder
       9. ☐ Post-traumatic stress disorder / trauma
       10. ☐ Schizophrenia / other psychotic disorder(s)
       11. ☐ Substance use disorder(s) / addiction
       12. ☐ Other (please specify): ________________
    3. ☐ No
    4. ☐ Prefer not to say
