# Supplementary material for: Mapping support systems: a cross-sectional examination of personal support networks, perceived support, mental health outcomes, and help-seeking behaviours among UK undergraduate students
Source: BMC Public Health. 2025 Sep 30;25:3190. doi: 10.1186/s12889-025-24360-1 (PMC12487519; doi:10.1186/s12889-025-24360-1)
Supplement: Supplementary file 2 — Supplementary Material 2. [file 12889_2025_24360_MOESM2_ESM.docx]

**Supplementary Tables**

**Table S1**

*Multiple linear regression predicting depression scores (N = 277; outliers included)*

| Predictor | *B* | β | 95% *CI* | *p* |
| --- | --- | --- | --- | --- |
| (Constant) | 14.50 |  | [10.99, 18.01] | < .001 |
| Female (ref = male) | -0.31 | -0.03 | [-1.44, 0.82] | .590 |
| Non-heterosexual (ref = heterosexual) | 1.73 | 0.14 | [0.33, 3.13] | .015 |
| Minority ethnic background (ref = white) | -0.51 | -0.05 | [-1.61, 0.59] | .361 |
| First-generation student | -0.14 | -0.02 | [-1.22, 0.93] | .793 |
| International student | -0.23 | -0.03 | [-1.32, 0.86] | .679 |
| Network size | 0.30 | 0.20 | [0.07, 0.53] | .012 |
| Mean relationship duration | 0.03 | 0.06 | [-0.04, 0.10] | .365 |
| Relationship diversity | -0.35 | -0.09 | [-1.02, 0.32] | .301 |
| Support diversity | 0.33 | 0.13 | [-0.06, 0.72] | .099 |
| Overall perceived support | -1.25 | -0.31 | [-1.79, -0.71] | < .001 |

*Note.* *B* = unstandardised coefficient, β = standardised coefficient.

**Table S2**

*Multiple linear regression predicting anxiety scores (N = 277; outliers included)*

| Predictor | *B* | β | 95% *CI* | *p* |
| --- | --- | --- | --- | --- |
| (Constant) | 12.65 |  | [9.64, 15.66] | < .001 |
| Female (ref = male) | 0.12 | 0.02 | [-0.84, 1.09] | .804 |
| Non-heterosexual (ref = heterosexual) | 0.22 | 0.02 | [-0.98, 1.42] | .721 |
| Minority ethnic background (ref = white) | -0.62 | -0.08 | [-1.56, 0.32] | .198 |
| First-generation student | 0.07 | 0.01 | [-0.85, 1.00] | .877 |
| International student | -0.35 | -0.04 | [-1.28, 0.59] | .465 |
| Network size | 0.18 | 0.14 | [-0.02, 0.38] | .077 |
| Mean relationship duration | 0.03 | 0.08 | [-0.02, 0.09] | .245 |
| Relationship diversity | -0.29 | -0.09 | [-0.86, 0.28] | .324 |
| Support diversity | 0.22 | 0.11 | [-0.12, 0.55] | .199 |
| Overall perceived support | -0.99 | -0.30 | [-1.46, -0.53] | < .001 |

*Note.* *B* = unstandardised coefficient, β = standardised coefficient.

**Table S3**

*Multiple linear regression predicting informal help-seeking (N = 277; outliers included)*

| Predictor | *B* | β | 95% *CI* | *p* |
| --- | --- | --- | --- | --- |
| (Constant) | 3.70 |  | [2.99, 4.42] | < .001 |
| Female (ref = male) | -0.22 | -0.11 | [-0.45, 0.01] | .060 |
| Non-heterosexual (ref = heterosexual) | -0.00 | 0.00 | [-0.29, 0.28] | .994 |
| Minority ethnic background (ref = white) | 0.16 | 0.09 | [-0.06, 0.39 | .157 |
| First-generation student | -0.18 | -0.09 | [-0.40, 0.04] | .116 |
| International student | -0.09 | -0.05 | [-0.32, 0.13] | .410 |
| Network size | 0.02 | 0.08 | [-0.02, 0.07] | .318 |
| Mean relationship duration | -0.00 | -0.03 | [-0.02, 0.01] | .654 |
| Relationship diversity | 0.05 | 0.07 | [-0.08, 0.19] | .444 |
| Support diversity | -0.11 | -0.22 | [-0.19, -0.03] | .009 |
| Overall perceived support | 0.15 | 0.19 | [0.04, 0.26] | .007 |

*Note.* *B* = unstandardised coefficient, β = standardised coefficient.

**Table S4**

*Multiple linear regression predicting formal help-seeking (N = 277; outliers included)*

| Predictor | *B* | β | 95% *CI* | *p* |
| --- | --- | --- | --- | --- |
| (Constant) | 3.25 |  | [2.55, 3.95] | < .001 |
| Female (ref = male) | -0.11 | -0.06 | [-0.34, 0.12] | .338 |
| Non-heterosexual (ref = heterosexual) | 0.09 | 0.04 | [-0.19, 0.37] | .534 |
| Minority ethnic background (ref = white) | 0.12 | 0.06 | [-0.10, 0.34] | .294 |
| First-generation student | -0.22 | -0.12 | [-0.44, -0.01] | .045 |
| International student | -0.14 | -0.08 | [-0.36, 0.08] | .215 |
| Network size | 0.00 | 0.01 | [-0.04, 0.05] | .931 |
| Mean relationship duration | -0.01 | -0.07 | [-0.02, 0.01] | .307 |
| Relationship diversity | -0.01 | -0.10 | [-0.14, 0.13] | .914 |
| Support diversity | 0.07 | 0.15 | [-0.01, 0.15] | .081 |
| Overall perceived support | -0.07 | -0.10 | [-0.18, 0.04] | .183 |

*Note.* *B* = unstandardised coefficient, β = standardised coefficient.
